# Meckel’s Diverticulum as a Rare Etiology of Small-Bowel Obstruction in an Otherwise Healthy Adult: A Case Report

**DOI:** 10.7759/cureus.105071

**Published:** 2026-03-11

**Authors:** David Hernandez, Dolores Ivkovic, Maryann H Saba, Marral K Pourmoghadam, Minkyung Kim, Frederick Tiesenga

**Affiliations:** 1 Medicine, St. George's University School of Medicine, St George's, GRD; 2 Medicine, St. George's University School of Medicine, St. George’s, GRD; 3 Medicine, St. George's University School of Medicine, St. George's, GRD; 4 General Surgery, West Suburban Medical Center, Chicago, USA

**Keywords:** gastro intestinal, meckel´s diverticulum, small-bowel obstruction, surgery general, torsion of meckel's diverticulum

## Abstract

Meckel’s diverticulum is the most common congenital anomaly of the gastrointestinal tract and is often asymptomatic in adults. When symptomatic, adult presentations frequently involve obstruction or inflammation and can be difficult to diagnose preoperatively due to nonspecific imaging findings. We report a case of a healthy 31-year-old male with progressive abdominal pain, distention, and emesis, ultimately found to have a high-grade small-bowel obstruction refractory to conservative management. Diagnostic laparoscopy identified a Meckel’s diverticulum tethered to the retroperitoneum at the transition point, and laparoscopic diverticulectomy relieved the obstruction. Histopathology demonstrated a true diverticulum without ectopic mucosa. This case highlights the importance of maintaining Meckel’s diverticulum in the differential diagnosis of unexplained small-bowel obstruction in younger adults and highlights the value of timely operative exploration when conservative therapy fails.

## Introduction

Meckel's diverticulum (MD) is one of the most prevalent congenital anomalies of the gastrointestinal tract arising from incomplete obliteration of the vitelline duct during embryonic development [1,2]. MD is a true diverticulum containing all layers of the intestinal wall and is located 2 feet (60-100 cm) from the ileocecal valve [2]. Although symptoms most commonly occur before two years of age, MD can become symptomatic at any age. Reported risk factors for symptomatic disease include male sex, age younger than 50 years, diverticulum length greater than 2 cm, ectopic tissue, and fibrous bands attached to the diverticulum [3]. When symptomatic, presentation is typically due to complications, often related to ectopic mucosa or inflammation. Gastrointestinal bleeding, commonly from peptic ulceration caused by ectopic gastric mucosa, is the primary manifestation within the pediatric population, whereas intestinal obstruction and diverticulitis are more prevalent in the adult population [2,4]. In adults, preoperative diagnosis can be challenging, and recent case reports emphasize that advanced imaging combined with a high index of suspicion may improve diagnostic accuracy, particularly in cases presenting with diverticulitis [5,6]. Complications may progress to perforation and sepsis if not promptly recognized and treated [1,2,5,7].

Surgical management remains the cornerstone of treatment for symptomatic MD. Laparoscopy is frequently favored because of its diagnostic utility, reduced morbidity, and faster postoperative recovery, although open laparotomy remains appropriate in select scenarios [8,9]. The operative approach, diverticulectomy versus segmental ileal resection, is guided by factors such as the presence of ectopic mucosa, inflammation at the base, and associated complications [2,5]. We present a case of a previously healthy 31-year-old Hispanic male who presented to the emergency department (ED) with acute abdominal pain, abdominal distention, and emesis.

## Case presentation

A 31-year-old Hispanic male with a surgical history of unspecified umbilical surgery in early childhood and no significant past medical history presented to the ED with abdominal pain, progressive distention, and emesis. Symptoms began on the morning of presentation with progressive abdominal bloating and mild intermittent periumbilical, suprapubic, and bilateral lower quadrant abdominal pain without relief from over-the-counter medications. He had one episode of emesis after drinking prune juice at home and a second episode of emesis in the ED. The patient’s last bowel movement occurred approximately 12 hours before presentation, and he had been unable to pass flatus since symptom onset.

On arrival, the patient appeared well-nourished and alert, but appeared to be in mild discomfort with an elevated blood pressure of 144/86 mmHg. Abdominal examination revealed abdominal distention with no significant abdominal scarring. The abdomen was soft with tenderness in the periumbilical, suprapubic, and bilateral lower quadrants without rebound or guarding. There were no palpable masses, and bowel sounds were present in all quadrants. The remainder of the physical examination was unremarkable.

Laboratory evaluation is shown in Table 1. A complete blood count (CBC), basic metabolic panel (BMP), and liver panel demonstrated leukocytosis [White blood cells (WBC) 16.6 x 10³/μl], erythrocytosis (7.08 x 106/µl), and mild hyperglycemia (137 mg/dL).

**Table 1:** Laboratory Results on Admission. Laboratory evaluation on admission, including a basic metabolic panel (BMP), liver panel, and complete blood count (CBC). Evaluation revealed leukocytosis of 16.6x10³/μl, erythrocytosis of 7.08x106/µl, glucose of 137 mg/dL, hematocrit of 46.2%, slightly elevated calcium of 10.5 mg/dL, and slightly elevated albumin of 5.2 g/dL.

Basic Metabolic Panel	Result	Reference range
Glucose	137 mg/dL	70-99 mg/dL
Blood urea nitrogen	14 mg/dL	7-25 mg/dL
Creatinine	1.01 mg/dL	0.6-1.3 mg/dL
Blood urea nitrogen/Creatinine ratio	14.0	6-20
Sodium	136 mmol/L	133-144 mmol/L
Potassium	4.1 mmol/L	3.5-5.1 mmol/L
Chloride	100 mmol/L	98-109 mmol/L
Carbon dioxide	26 mmol/L	21-31 mmol/L
Calcium	10.5 mg/dL	8.6-10.3 mg/dL
Liver Panel	Result	Reference range
Aspartate aminotransferase	24 U/L	10-40 U/L
Alanine aminotransferase	38 U/L	7-56 U/L
Alkaline phosphatase	72 U/L	35-104 U/L
Total bilirubin	0.6 mg/dL	0.0-1.0 mg/dL
Albumin	5.2 g/dL	3.5 - 5.0 g/dL
Complete Blood Count	Result	Reference
White blood cell	16.6 x 10³/μl	4.0-11 x 10³/μl
Red blood cell	7.08 x 10^6^/µl	4.2-5.9 x 10^6^/μl
Hemoglobin	14.8 g/dL	13.5-17.5 g/dL
Hematocrit	46.2%	34.7-45.1%

**Figure 1:**
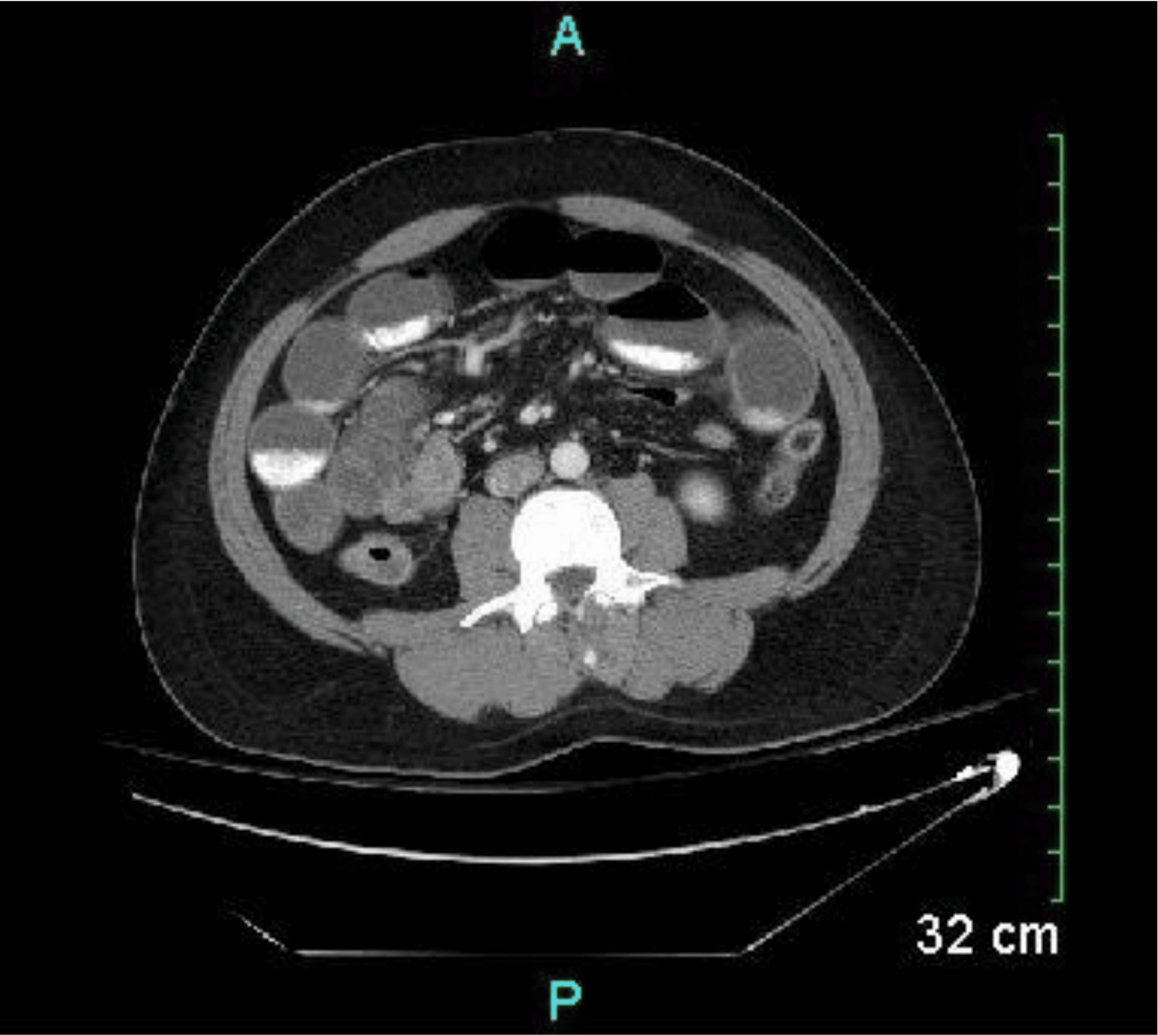
CT Scan of Abdomen and Pelvis with Contrast. CT scan of the abdomen and pelvis with contrast showing a partial small-bowel obstruction characterized by dilated loops of small bowel throughout the abdomen, as well as enteritis characterized by wall thickening involving multiple loops of small bowel.

A CT scan of the abdomen and pelvis with contrast was performed to investigate a possible small-bowel obstruction (SBO) (Figure 1). The CT demonstrated diffusely dilated small bowel loops measuring up to 3.8 cm in the left hemi-abdomen, with scattered areas of small bowel wall thickening, more pronounced in the right hemi-abdomen. These findings were suggestive of infectious or inflammatory enteritis with superimposed partial small-bowel obstruction, and no discrete transition point was identified.

The patient was admitted, and initial management included bowel rest, placing the patient on nothing by mouth (NPO), isotonic intravenous (IV) fluids to prevent dehydration, and placing a nasogastric (NG) tube for gastric decompression. During the first eight hours, approximately 300 mL of bilious output was obtained via NG suction. The patient was started on IV pantoprazole 40 mg daily for stress ulcer prophylaxis and empiric broad-spectrum IV piperacillin-tazobactam per infectious disease recommendations for possible infectious or inflammatory enteritis. Electrolytes, CBC, and lactate levels were serially monitored to assess for metabolic derangements or early signs of bowel ischemia.

**Figure 2:**
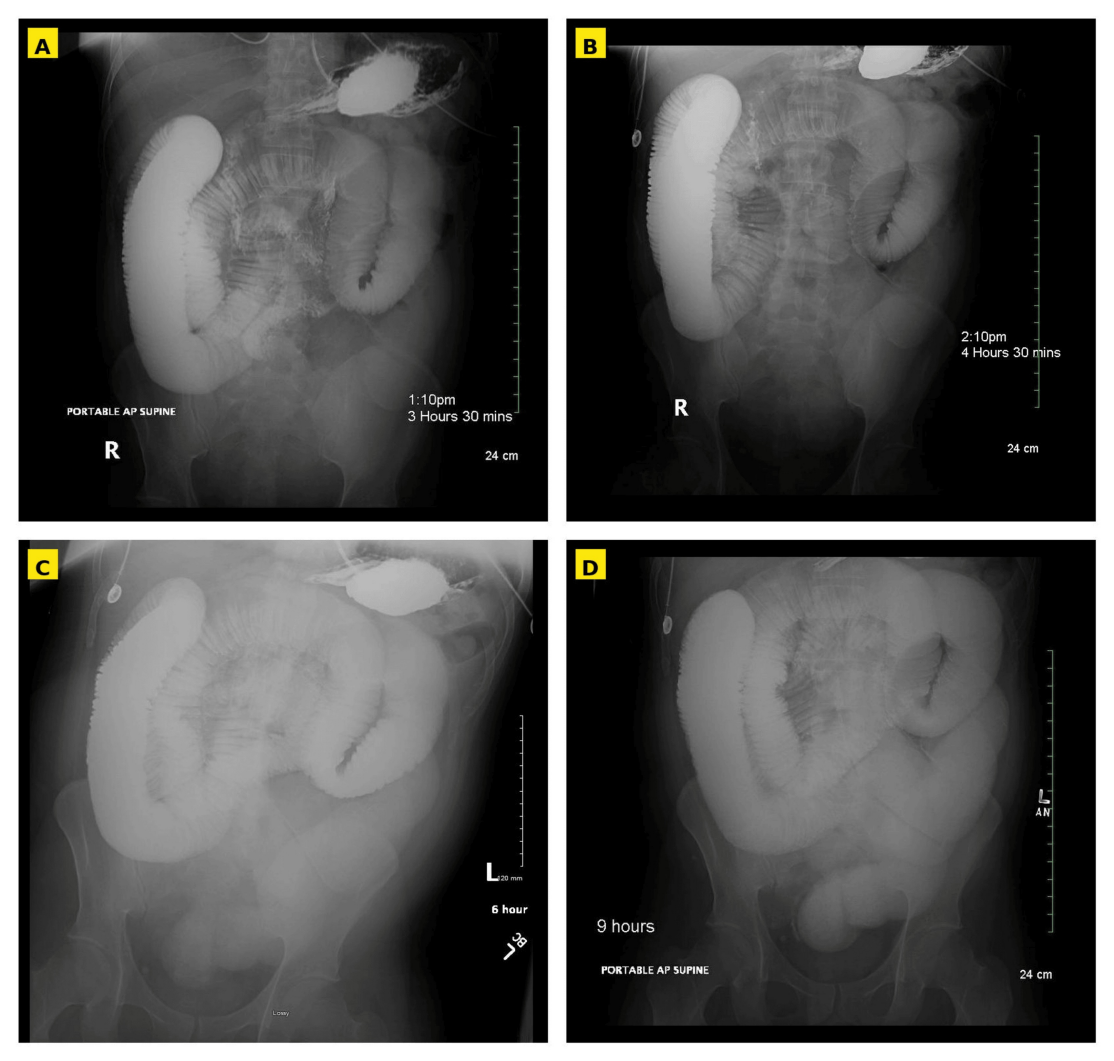
Fluoroscopic Small-Bowel Follow-Through. Abdominal radiographs obtained at A. 3.5 hours, B. 4.5 hours, C. 6 hours, and D. 9 hours after administration of fluoroscopic contrast. At 3.5 hours, contrast remained within the dilated small bowel without progression into the colon, unchanged at the 6-hour film, and no further progression was seen between the 6-hour and 9-hour film.

After 48 hours of conservative management, the patient’s abdominal distention persisted, and he continued to report abdominal discomfort without passage of flatus or stool. A fluoroscopic small-bowel follow-through (SBFT) was performed, which demonstrated dilated small bowel containing contrast with no contrast seen within the colon (Figure 2). Radiographic imaging at nine hours following contrast administration showed no interval progression compared with the six-hour film, raising concern for a high-grade obstruction and prompting surgical intervention.

**Figure 3:**
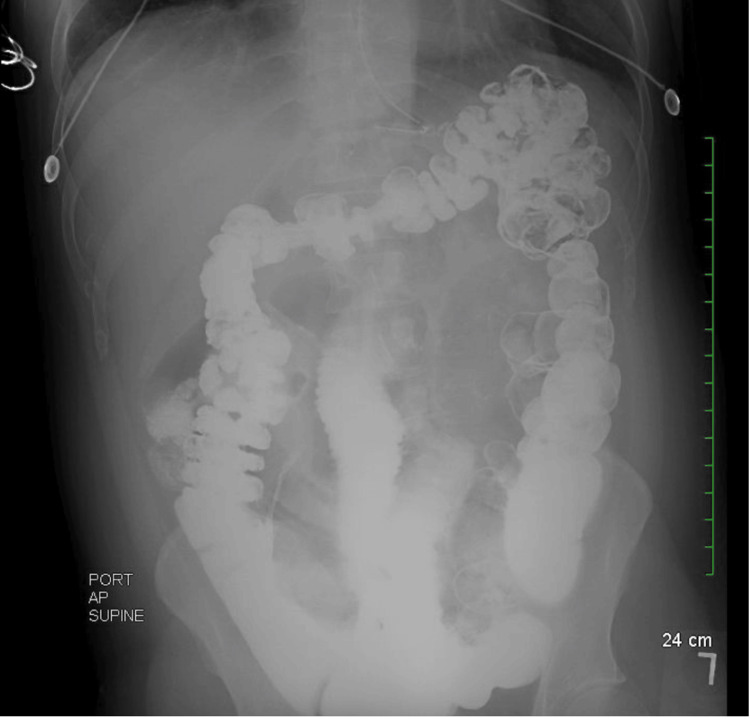
Abdominal X-Ray 24 Hours Post Small-Bowel Follow-Through. An Abdominal x-ray was obtained for comparison with the small bowel follow-through from the previous day. Contrast now projects over the colon. A few distended small bowel loops, which have contrast intraluminally, are still visualized, measuring up to 5 cm in diameter. The NG tube is visualized within the stomach lumen.

A repeat abdominal radiograph obtained the morning of surgery demonstrated persistently dilated small-bowel loops measuring up to 5 cm with the NG tube appropriately positioned within the stomach (Figure 3).

Although passage of contrast into the colon within 24 hours of a small-bowel follow-through can predict nonoperative resolution, the absence of contrast progression together with persistent abdominal pain and distention despite gastric decompression supported operative intervention [10].

The patient underwent a diagnostic laparoscopy for definitive management. Intraoperatively, markedly dilated proximal small bowel loops were visualized. The distal ileum and ileocecal valve appeared collapsed, and a clear transition point was identified where a pouch-like structure was tethered to the retroperitoneum. This structure was consistent with MD, which was determined to be the source of obstruction. The diverticulum was carefully dissected and resected using a stapling device, and a mesenteric soft-tissue mass was excised and sent for histopathologic evaluation. No evidence of perforation, ischemia, or necrosis was observed. A Jackspn-Pratt (JP) drain was placed in the right lower quadrant, and all surgical ports were closed without complications.

Postoperatively, the patient was transferred to the surgical ward for observation and supportive care. He was maintained on IV fluids and continued on his antibiotic regimen until postoperative day three. The NG tube was removed once evidence ofbowel function returned, as indicated by the passage of flatus and stool. His diet gradually advanced from clear liquids to a soft, low-fiber diet, which he tolerated well. Serial laboratory evaluations demonstrated normalization of his leukocytosis from 16.6×10³/μL to 9.9×10³/μL (Table 1).

The patient’s postoperative recovery was uneventful. He reported complete resolution of abdominal pain and distention, with no further episodes of nausea or vomiting. The JP drain was removed before discharge after minimal serosanguinous output. The patient was discharged home in stable condition with instructions to follow up in the outpatient surgery clinic within two weeks.

**Figure 4:**
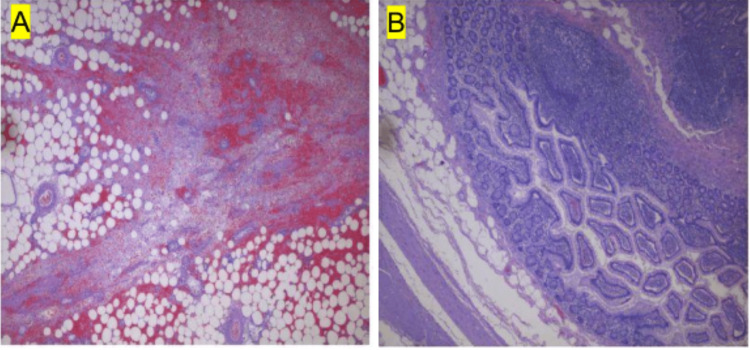
Photomicrograph of a Biopsy of A. Intra-Abdominal Mass and B. Meckel’s Diverticulum. Figure 4A is a photomicrograph of soft tissue from the intra-abdominal mass excised from the mid small bowel. The histology shows fibroadipose tissue with focal acute inflammation, fat necrosis, vascular congestion, hemorrhage, and reactive change. Figure 4B demonstrates diverticular tissue from the distal ileum containing all layers of the intestinal wall, without ectopic mucosa and with no evidence of dysplasia or malignancy.

Histopathological analysis of the resected specimen confirmed the diagnosis of MD, showing a true diverticular structure containing all layers of the intestinal wall without evidence of ectopic gastric or pancreatic mucosa (Figure 4).

## Discussion

Although MD typically presents in childhood, it may remain asymptomatic into adulthood and later manifest through complications such as gastrointestinal bleeding or intestinal obstruction [1,5]. In adults, intestinal obstruction is the most frequently observed complication that may occur through several mechanisms, including volvulus around a fibrous band, diverticular intussusception, incarceration within a hernia sac, or inflammation leading to luminal narrowing [2,8,11]. In our patient, a high-grade SBO caused by MD being tethered to the retroperitoneum, a mechanism consistent with prior reports of mechanical obstruction related to MD [5,11]. This case highlights the importance of maintaining a high index of suspicion for MD in adults presenting with unexplained SBO, particularly younger patients without a significant history of abdominal surgery.

While a CT is sensitive for detecting intestinal obstruction, it often underperforms in determining the underlying etiology or directly visualizing the diverticulum, especially when findings are nonspecific [9,12]. In this case, CT revealed a diffuse small-bowel dilation with mural thickening and no distinct transition point, giving the impression of possible enteritis with partial SBO rather than MD. Although the gold standard imaging technique, technetium-99m pertechnetate scintigraphy, also known as a Meckel scan, remains critical in pediatric patients for diagnosis, its sensitivity decreases in adults, in part due to reduced uptake in ectopic gastric mucosa and variability in the presence of ectopic tissue [12,13]. Although newer modalities, including high-resolution CT protocols and capsule endoscopy, may improve preoperative recognition, definitive diagnosis in adults often still depends on intraoperative identification and histopathologic confirmation [6,12,13]. Prior studies have emphasized the combination of advanced imaging with a strong clinical suspicion to obtain a timely diagnosis and surgical intervention [5,6]. Despite evolving imaging techniques, MD remains challenging to diagnose preoperatively because radiologic findings are frequently nonspecific [9,12,13].

The differential diagnosis for this presentation included gastritis and SBO unrelated to MD, as these entities can present with overlapping features such as abdominal pain, emesis, and bowel wall thickening on imaging (Table 2) [14,15].

**Table 2:** Comparative Clinical Features of Meckel’s Diverticulitis and Differential Diagnoses. Comparison of Meckel's diverticulitis with other potential differential diagnoses presenting with acute abdominal pain. Although several conditions may share overlapping gastrointestinal manifestations, focal inflammatory changes in a blind-ending small bowel pouch on CT imaging remain characteristic of Meckel’s diverticulitis. GI: gastrointestinal

Condition	Typical Presentation	Similarity to Meckel’s Diverticulitis	Key Differences from Meckel’s Diverticulitis
Inflamed Meckel’s diverticulum (Meckel’s diverticulitis)	Acute right lower quadrant or periumbilical pain, nausea, emesis, and sometimes GI bleeding [11].	-	CT: blind-ending pouch with wall thickening, surrounding inflammation, and possible localized fluid or air if perforated [11].
Gastritis	Epigastric pain, nausea, emesis, anorexia; may have melena if bleeding occurs; often subacute or chronic. [14]	Both may present with emesis and epigastric pain.	Gastritis is localized to the stomach, lacks signs of peritonitis or focal diverticular inflammation, and does not produce a blind-ending pouch on imaging. CT: normal or mild gastric wall thickening. [14]
Small-Bowel Obstruction	Crampy abdominal pain, emesis (may be feculent), distension, obstipation, and high-pitched bowel sounds. [15]	Both may present with nausea, emesis, and abdominal pain.	SBO typically features mechanical obstruction signs and a clear transition point; it lacks localized inflammation or diverticular outpouching, as seen in Meckel’s diverticulitis. CT: dilated proximal bowel loops, transition point, collapsed distal bowel. [15]

The distinguishing feature of MD relies on the presence of a blind-ending pouch arising from the small intestine, associated with localized inflammation and mechanical obstruction [11]. Recognition of imaging findings such as these, in conjunction with presenting or deteriorating symptoms despite conservative management, prompts surgical exploration. This case reinforces the consideration of MD in the differential diagnosis of SBO in adults, where congenital anomalies are less frequently suspected. Timely laparoscopic intervention can prevent complications such as ischemia or perforation, which underscores the clinical significance of MD as a rare but critical cause of SBO in adults [1,2,4,5,8,11].

Surgical management is the definitive treatment for symptomatic MD, with options including diverticulectomy or segmental ileal resection depending on factors such as the presence of ectopic mucosa, inflammation, or perforation [2,8,9]. Laparoscopy is often favored due to diagnostic utility, minimal invasiveness, and favorable postoperative outcomes compared with open approaches [8,9]. Laparoscopy-assisted diverticulectomy is an additional minimally invasive option in which the diverticulum and adjacent ileum are exteriorized through a small incision for direct palpation and extracorporeal resection. This may be useful when ectopic mucosa is suspected, or the diverticular base is broad, because purely laparoscopic diverticulectomy can make it difficult to confirm complete excision of ectopic tissue and determine whether segmental ileal resection is warranted [16]. In our patient, laparoscopic management relieved the obstruction and was followed by an uncomplicated recovery and symptom resolution. Histopathology demonstrated MD without ectopic mucosa or perforation, supporting a favorable prognosis and confirming the adequacy of diverticulectomy as definitive therapy in this setting.

## Conclusions

MD is an uncommon but important cause of SBO in otherwise healthy adults. In this case, ongoing obstructive symptoms with a nondiagnostic CT prompted additional evaluation, and an SBFT demonstrated high-grade obstruction. Diagnostic laparoscopy revealed a Meckel’s diverticulum tethered to the retroperitoneum at the transition point, and laparoscopic diverticulectomy led to an uncomplicated postoperative course with pathology confirming a true diverticulum. Clinicians should consider Meckel’s diverticulum when a healthy adult has unexplained small-bowel obstruction that does not resolve with conservative therapy. Timely operative exploration can both confirm the diagnosis and prevent complications such as ischemia or perforation.

## References

[1] Sagar J, Kumar V, Shah DK (2006). Meckel's diverticulum: a systematic review. J R Soc Med.

[2] Hansen CC, Søreide K (2018). Systematic review of epidemiology, presentation, and management of Meckel's diverticulum in the 21st century. Medicine (Baltimore).

[3] An J, Zabbo CP (2023). Meckel diverticulum. https://www.ncbi.nlm.nih.gov/books/NBK499960.

[4] Hong SN, Jang HJ, Ye BD (2016). Diagnosis of bleeding Meckel's diverticulum in adults. PLoS One.

[5] Hernández JD, Valencia G, Girón F (2023). Meckel's diverticulum: analysis of 27 cases in an adult population. Front Surg.

[6] Le Nguyen TT, Nguyen VT, Tong HD (2024). Preoperative diagnosis of Meckel diverticulitis with and without perforation in adult: two case reports. Int J Surg Case Rep.

[7] Weerakkody Y, Spires R, Silverstone L (2025). Meckel diverticulum. Reference article. Radiopaedia.org.

[8] Blouhos K, Boulas KA, Tsalis K (2018). Meckel's diverticulum in adults: surgical concerns. Front Surg.

[9] Hosn MA, Lakis M, Faraj W, Khoury G, Diba S (2014). Laparoscopic approach to symptomatic meckel diverticulum in adults. JSLS.

[10] Abbas S, Bissett IP, Parry BR (2007). Oral water soluble contrast for the management of adhesive small bowel obstruction. Cochrane Database Syst Rev.

[11] Jabeen N, Abdulla HA, Alqaseer A (2021). Meckel’s diverticulum: a rare cause of small bowel obstruction in an adult. International Surgery Journal.

[12] Farrell MB, Zimmerman J (2020). Meckel's diverticulum imaging. J Nucl Med Technol.

[13] Titley-Diaz WH, Aziz M (2025). Meckel scan. https://www.ncbi.nlm.nih.gov/books/NBK560500/.

[14] Rugge M, Sugano K, Sacchi D (2020). Gastritis: an update in 2020. Current Treatment Options in Gastroenterology.

[15] Rami Reddy SR, Cappell MS (2017). A systematic review of the clinical presentation, diagnosis, and treatment of small bowel obstruction. Curr Gastroenterol Rep.

[16] Altinli E, Pekmezci S, Gorgun E (2002). Laparoscopy-assisted resection of complicated Meckel's diverticulum in adults. Surgical Laparoscopy, Endoscopy & Percutaneous Techniques.

